# Application of exosomes in the diagnosis and treatment of pancreatic diseases

**DOI:** 10.1186/s13287-022-02826-y

**Published:** 2022-04-08

**Authors:** Li Han, Zhirong Zhao, Ke Yang, Mei Xin, Lichen Zhou, Siping Chen, Shibo Zhou, Zheng Tang, Hua Ji, Ruiwu Dai

**Affiliations:** 1 General Surgery Center, General Hospital of Western Theater Command, Chengdu, 610083 Sichuan Province China; 2 College of Medicine, Southwest Jiaotong University, Chengdu, 610031 China; 3 Department of Cardiovascular Surgery, General Hospital of Western Theater Command, Chengdu, 610083 Sichuan Province China; 4 College of Clinical Medicine Southwest, Medical University, Luzhou, 646000 Sichuan Province China

**Keywords:** Exosome, Pancreatic disease, Diagnosis, Apoptosis, Immune regulation, Angiogenesis, Cell migration and proliferation

## Abstract

Pancreatic diseases, a serious threat to human health, have garnered considerable research interest, as they are associated with a high mortality rate. However, owing to the uncertain etiology and complex pathophysiology, the treatment of pancreatic diseases is a challenge for clinicians and researchers. Exosomes, carriers of intercellular communication signals, play an important role in the diagnosis and treatment of pancreatic diseases. Exosomes are involved in multiple stages of pancreatic disease development, including apoptosis, immune regulation, angiogenesis, cell migration, and cell proliferation. Thus, extensive alterations in the quantity and variety of exosomes may be indicative of abnormal biological behaviors of pancreatic cells. This phenomenon could be exploited for the development of exosomes as a new biomarker or target of new treatment strategies. Several studies have demonstrated the diagnostic and therapeutic effects of exosomes in cancer and inflammatory pancreatic diseases. Herein, we introduce the roles of exosomes in the diagnosis and treatment of pancreatic diseases and discuss directions for future research and perspectives of their applications.

## Background

Pancreatic diseases, such as acute pancreatitis (AP), chronic pancreatitis (CP), pancreatic cancer (PaCa), and diabetes, form a large category of complex diseases with high incidence and mortality. The global mortality rates associated with AP and CP are 1.60 and 0.09 per 100 000 individuals per year, respectively [1]. AP is the fifth most common cause of death of patients in hospitals, with an incidence rate of 34 per 100 000 individuals per year globally [2–5]. Approximately 36% of CP is transformed from recurrent AP, and its incidence is higher in middle-aged and elderly men [6]. Similar to inflammatory pancreatic diseases, PaCa and diabetes are also serious diseases with an extremely poor prognosis. PaCa remains one of the deadliest cancers worldwide, with a five-year survival rate of as low as 6% [7]; diabetes affects around 425 million people per year globally [8]. Pancreatic diseases have garnered attention, especially their early-stage diagnosis and effective therapies. Currently, the widely used diagnosis methods for most pancreatic diseases are mostly based on serum biochemical markers and abdominal imaging. However, digestive enzymes that are commonly used to diagnose pancreatitis (amylase and lipase) have low sensitivity and specificity [9]. With respect to the treatment of AP and CP, supportive and symptomatic therapy, including analgesics and fluid resuscitation, is commonly used [10, 11]. This approach may relieve symptoms in a short time but does not address the specific pathogenesis. Neoadjuvant chemotherapy and surgical resection are primarily used as treatment strategies for PaCa, but the prognosis is still poor owing to the high recurrence rate [12, 13]. Considering that both development of detection methods based on serological techniques and the exploration of targeted therapy are in early stages, it is of importance to perform additional extensive and in-depth investigations on diagnosis and treatment of pancreatic diseases.

Exosomes, an important carrier of intercellular communication signals, play important roles in the diagnosis and treatment of pancreatic diseases. Exosomes contain various proteins, RNAs, and other components that regulate the inflammatory response, angiogenesis, and cell protection after injury by influencing gene expression at the mRNA level [14–16]. Exosomes participate in various physiological and pathological processes such as biological development, epigenetic regulation, immune regulation, and tumor occurrence and progression [17–20]. It has been proved that exosomes are involved in several stages of pancreatic disease development, such as apoptosis, immune regulation, angiogenesis, cell migration, and cell proliferation. Therefore, extensive changes in the number and type of exosomes may indicate an abnormal biological behavior of pancreatic cells. This phenomenon could be exploited for the development of exosomes as a new biomarker or therapeutic strategy. Owing to their high sensitivity and specificity, exosomes have been used in the diagnosis and treatment of cancer and inflammatory diseases, especially in the diagnosis of tumors that are difficult to detect in the early stages of routine physical examination [21, 22]. In addition, the changes in some contents in the exosomes may indicate the evolutionary trend of the disease. Monitoring these changes to guide clinical diagnosis and treatment will help physicians understand the conditions of patients and adjust their diagnosis and treatment plan in time. Therefore, exosomes are becoming a new tool to gain specific information of diseases; moreover, they can serve as biomarkers for pathophysiology [23, 24]. In summary, studies have shown the potential of exosomes for multiple applications in the treatment of pancreatic diseases.

In this review, we focus on the role of exosomes as specific biomarkers for the diagnosis of various pancreatic diseases. We further introduce the role of exosomes in the treatment of pancreatic diseases, including apoptosis and immune regulation, angiogenesis, cell migration, and cell proliferation. We summarize the trends and challenges of future research and the application of exosomes in pancreatic diseases.

## Exosomes: biology, identification, and isolation

Among the extracellular vesicles released from mammalian cells (namely, exosomes, microvesicles, and apoptotic vesicles), exosomes are the most broadly investigated. Exosomes originate from the membranes of multivesicular bodies; they appear as double-layer phospholipid membrane structures in electron microscopy, of diameters ranging from 40 to 150 nm [25]. Under physiological or pathological conditions, exosomes can transfer bioactive molecules such as DNA, RNAs, and proteins locally or remotely from donor cells to recipient cells [26]. In addition, exosomes can be easily manipulated and wrapped in exosome biogenesis in donor cells, and they can cross a variety of biological barriers [27]. They are classified based on size, density, morphology, and the presence of common surface markers such as CD63, CD81, and CD9 tetraspanins, fusion proteins, endosome-associated proteins, and heat shock proteins [28].

Current studies have mainly extracted and identified exosomes from cell culture media and biological fluids [29]. After ultracentrifugation (100 000 g), relatively pure exosomes can be isolated using an additional sucrose gradient step, with exosomes at a sucrose density of 1.13–1.19 g/mL [30]. In addition, several commercial kits are based on the principle of polymer precipitation to separate exosomes, and the purification efficiency of different kits is different [31]. The polymer precipitation method has the advantages of simplicity and rapidity; moreover, it does not require the use of expensive equipment. However, commercialized kits are expensive, and exosomes could get contaminated by polymers in the purification process, which will affect the subsequent experiments [32].

## Application of exosomes in the diagnosis of pancreatic diseases

### Diagnosis of AP

**Fig. 1:**
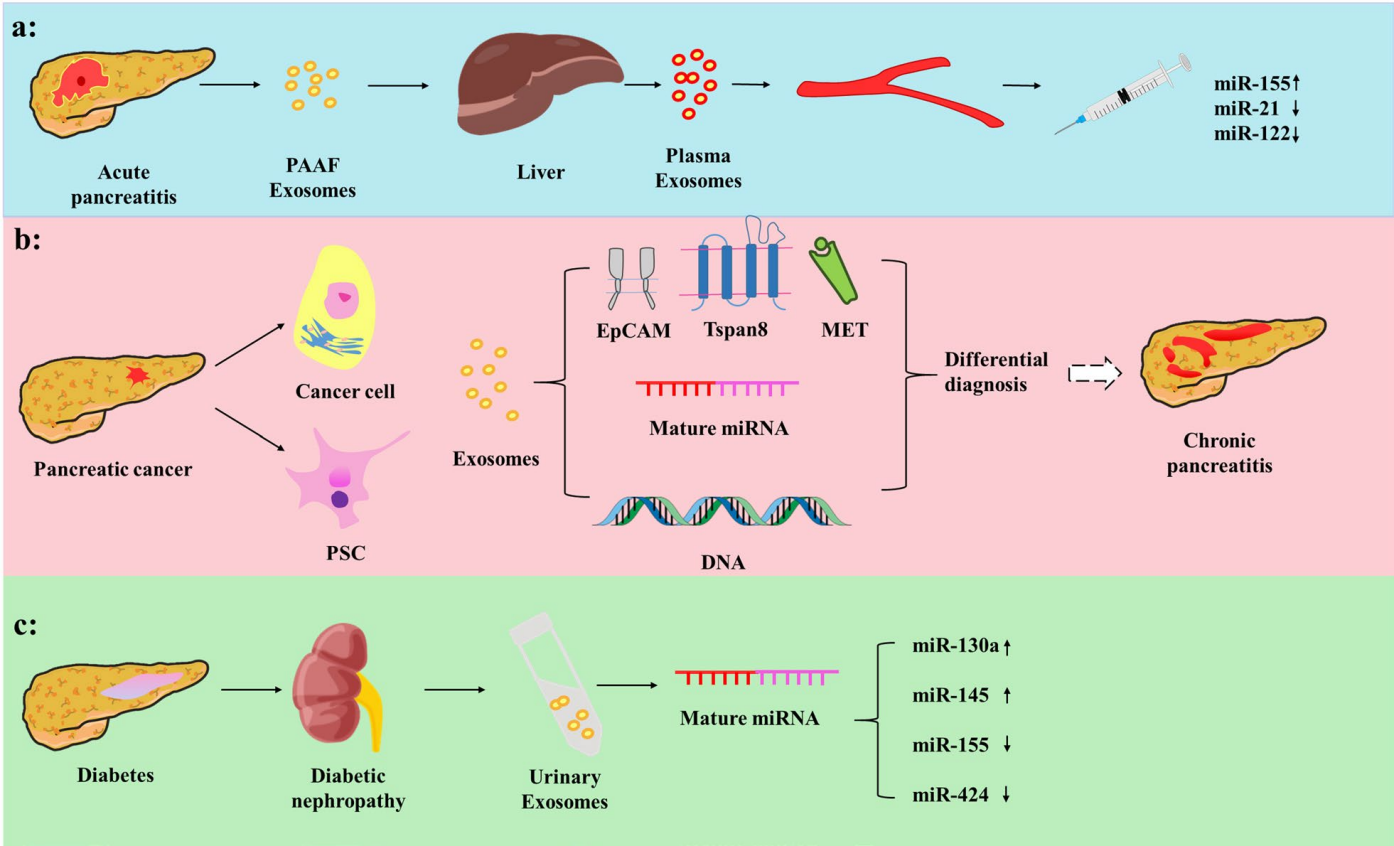
Application of exosomes in the diagnosis of pancreatic diseases. **a** The miRNA expressed in plasma-derived exosomes display greater diagnostic value in AP. **b** Exosomes may be highly specific in the identification of CP and PaCa. **c** Exosomes from plasma are significant for early diagnosis of diabetes

The timely and effective diagnosis of AP is key to its treatment. The severity of AP correlates with the inflammatory capacity of exosomes in the early stages of the disease. Exosomes isolated from the blood of patients with mild pancreatitis had no significant effect on inflammatory cells, whereas exosomes from patients with severe pancreatitis triggered nuclear transcription factor-κB (NF-κB) activation, tumor necrosis factor (TNF)-α release, and other inflammatory processes [33]. Currently, several studies have focused on the role of microRNA (miRNA) carried by exosomes in the diagnosis of systemic inflammation caused by AP (Fig. 1a). In a previous study, exosomal miRNAs were extracted and examined using microarrays; 30 miRNAs were found to be upregulated in AP [1]. Furthermore, the study demonstrated that pancreatic acinar cells regulate macrophage activation by secreting exosomes carrying miRNAs. Additionally, target genes of differentially expressed miRNAs were found to regulate macrophage activation via the TRAF6-TAB2-TAK1-NIK/IKK-NF-κB pathway. Other studies have confirmed that in AP, two different exosome populations are produced, with apparent differences in their origin, tissue distribution, molecular content, and physiological effects. Pancreatitis plasma exosomes, compared to pancreatitis-associated ascitic fluid (PAAF) exosomes, are enriched in inflammatory miR-155 and express low levels of miR-21 and miR-122 [34]. In addition, plasma exosomes have higher pro-inflammatory activity in macrophages than PAAF exosomes. The plasma miRNA expression profile in patients with AP has been established, and a prospective study including a larger number of samples is in progress. The detection of exosomes carrying miR presents an optimistic prospect to diagnose AP [35].

### Diagnosis of CP

There is a lack of effective diagnostic markers for CP. In clinical practice, CP is sometimes misdiagnosed as pancreatic ductal adenocarcinoma (PDAC), resulting in unnecessary pancreatectomy [36]. Distinguishing PDAC from CP based on miRNA expression may be a future research direction (Fig. 1b). It has been reported that high exosomal levels of miRNA-10b, miR-21, miR-30c, and miR-181a, and low miR-let7a can be readily used to differentiate PDAC from normal control and CP samples. Furthermore, the exosomal miRNA signature is better than exosomal GPC1 or plasma CA 19-9 levels when establishing a diagnosis of PDAC and distinguishing PDAC from CP [22]. A proof-of-concept study showed that exosomal DNA can be used to distinguish PDAC from CP [37]. It demonstrated the potential clinical utility of circulating exosomal DNA to identify KRAS and TP53 mutations in patients with pancreas-associated pathologies, including PDAC and CP, and healthy human subjects. In 48 patients with PDAC, digital PCR analyses of exosomal DNA identified the KRAS mutations in 39.6% of patients. Exosomes may be highly specific for the identification of CP and PaCa.

### Diagnosis of PaCa

Exosomes play important roles in cancer genesis, cancer-related immune reactions, and metastasis. They also have potential as novel biomarkers for the early detection of PaCa. Evaluation of exosomal proteins and miRNAs in blood samples from patients with PaCa, CP, benign pancreatic tumors, non-PaCa, and healthy volunteers has revealed excellent sensitivity for PaCa compared with all other groups (Fig. 1b). Significant differences were observed in exosomal protein markers CD44v6, Tspan8, EpCAM, MET, and CD104, and combinations of miR-1246, miR-4644, miR-3976, and miR-4306, in both serum exosomes and exosome-depleted serum [38, 39]. Certain proteins, such as EGFR, can be released in distinct isoforms from PaCa in exosomes, suggesting that exosomes may be involved in mediating intercellular communication and may be used as potential biomarkers [40]. Another study found that pancreatic stellate cell (PSC)-derived exosomes stimulate the proliferation, migration, and expression of miRNAs of chemokine ligands in PaCa cells. This study determined the miRNA expression profile in PSC-derived exosomes, which may play a role in the interactions between PSCs and PaCa cells [41].

Exosomes have several advantages as biomarkers. First, exosomes act as messengers of original cells and are detectable in a variety of body fluids, including blood, saliva, and urine [42–44]. Second, exosomes are more abundant in patients with cancer than in health controls, which can be attributed to the tumor microenvironment that can promote exosome release [45]. Third, when the tumor mass reached a substantial volume or advanced stage, its content type of exosome messengers differs from that in early stage, suggesting that exosomes may play a role in tumor staging diagnosis [46]. Finally, clinicians may treat human cancer by modulating exosome contents [47, 48]. The detection of serum exosomes in patients with pancreatic tumors is a hot research topic, as this may translate to the application of exosomes in pancreatic disease diagnosis and treatment. Therefore, clinical trials on pancreatic tumor screening technology based on serum exosome detection may provide compelling data in the near future.

### Diagnosis of diabetes and its complications

Recent studies have shown that exosomes can be used as biomarkers for metabolic diseases, thereby detecting disease risk markers to prevent disease or provide possible treatments. Therefore, it is important to identify patients at a risk of diabetes and promote an early diagnosis of diabetes (Fig. 1c). A study involving cross-sectional and longitudinal groups of participants with normal blood glucose and those with prediabetes or diabetes verified that circulating exosome levels in patients with Type 2 diabetes were significantly higher than those in participants with normal blood glucose [49]. In a previous study, exosomes were separated from the urine samples of rats through differential analysis of the renal proteome in an early diabetic nephropathy (DN) rat model, and then protein expression in response to substantial changes in DN in rat tissues to human kidney tissues and urine exosomes was compared. It was concluded that the expression of regulcin protein in the renal tissues of subjects with DN decreases. This significant change was also verified in exosomes isolated from rat urine. This suggests that urinary exosomes can be used as a novel tool for the early diagnosis and monitoring of DN progression [50]. Other studies have analyzed the urine levels of AQP5 and AQP2 excreted by exosomes in 35 patients with diabetes, suggesting that exosomal AQP5 and AQP2 may be used as novel noninvasive biomarkers to help classify the clinical stages of DN [51]. With further research development, urinary exosomes can be used for the early diagnosis of diseases, with miRNA contained in exosomes playing a role. For example, one study assessed miRNA expression in urinary exosomes of type-1 diabetes mellitus (T1DM) patients with and without early DN. The results showed that miR-130a and miR-145 were enriched in urine exosomes of patients with microalbuminuria, whereas miR-155 and miR-424 were decreased. This study verified that the level of urinary exosomal miRNA changes in T1DM patients with early DN, and miR-145 may represent a novel biomarker for diabetic complications [52].

## Application of exosomes in the treatment of pancreatic diseases

### Exosome-based treatment via apoptosis regulation

**Fig. 2:**
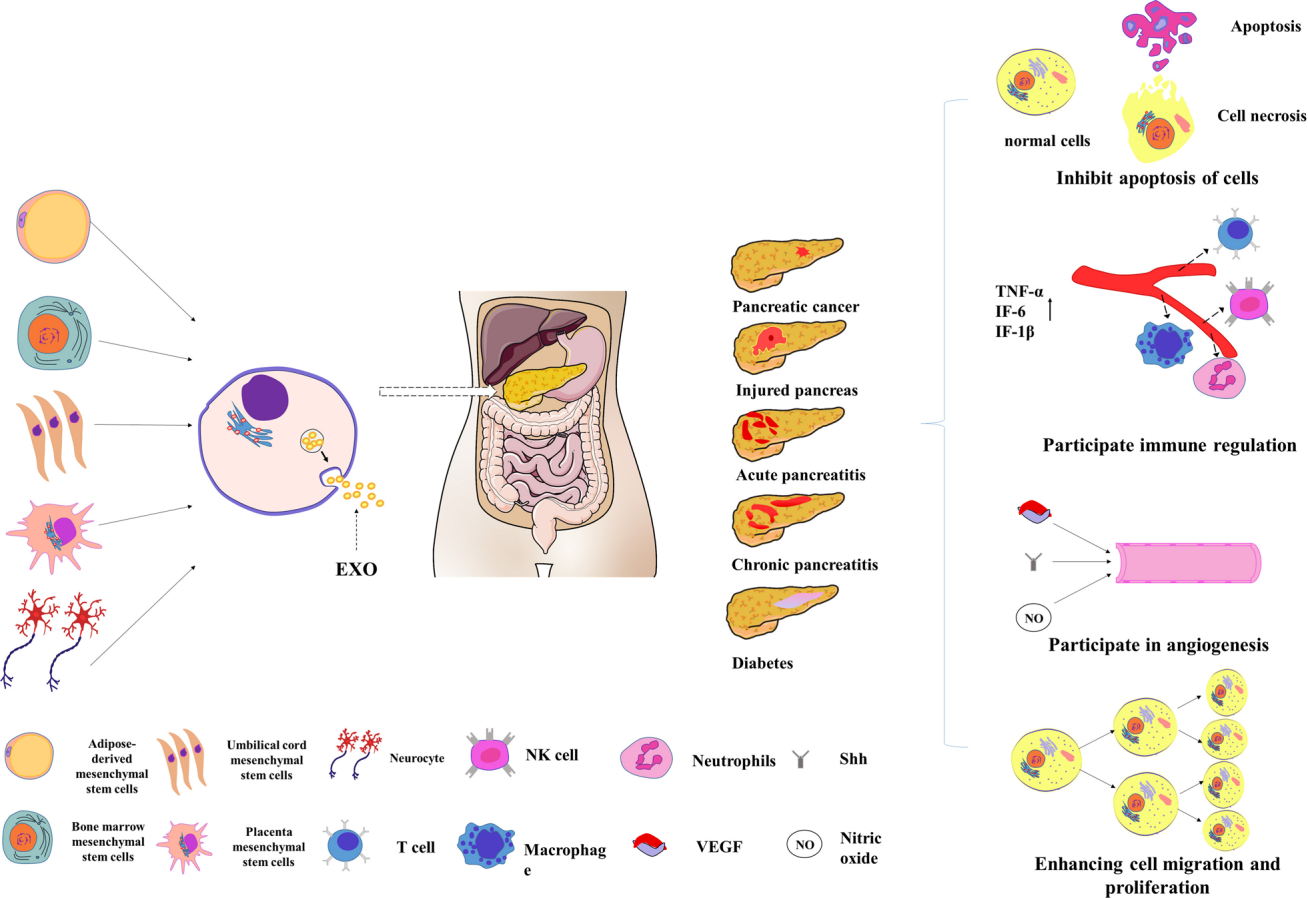
Application of exosomes in the treatment of pancreatic diseases. Exosomes from various cell types use different mechanisms to treat pancreatic diseases, including apoptosis, immune regulation, angiogenesis, cell migration, and proliferation

Apoptosis is characterized by mitochondrial leakage and dysfunction as well as the activation of apoptotic signaling pathways. Recent studies have shown that apoptosis can be mediated by exosomes through various signaling pathways such as the Wnt/β-catenin, NF-κB, and Akt/PI3K signaling pathways [53]. The occurrence and development of pancreatic diseases are closely related to exosome-mediated apoptosis (Fig. 2) [54, 55]. Exosomes secreted by bone marrow mesenchymal stem cells (BMSCs) can regulate inflammatory cytokines and reduce the infiltration of inflammatory cells through the NF-κB signaling pathway. As a result, serum lipase and amylase levels decrease, indicating the repair and regeneration of necrotic pancreatic tissues in AP [56]. Exosomes affect pancreatitis by regulating various signaling pathways. In addition, exosomes may affect other types of pancreatic diseases such as diabetes and PaCa. A previous study successfully established a rat model of Type 2 diabetes mellitus (T2DM) using a high-fat diet combined with streptozotocin and confirmed that human mesenchymal stem cell-derived exosomes inhibit the apoptosis of rat β-cells to alleviate T2DM in rats [57]. In the treatment of PDAC, exosomes can be used as miRNA loaders to significantly reduce the growth of transplanted tumors in animal models by inhibiting the proliferation or invasion of PDAC and increasing apoptosis and cell cycle arrest [58, 59].

The mechanism underlying exosome involvement in apoptosis in various pancreatic diseases by regulating different signaling pathways has been studied extensively, but the specific molecular mechanisms and targets remain unclear. The mechanism and application of exosomes in apoptosis should be studied further.

### Exosome-based treatment via immune regulation

Recent studies have shown that exosomes contain a variety of immune-related molecules, including lysosome-associated membrane proteins and major histocompatibility complex class I and II molecules, which can stimulate macrophage polarization and T cell polarization to anti-inflammatory regulatory T cells (Treg), and inhibit the proliferation of inflammatory CD4+ T, CD8+ T, and natural killer cells [60–64]. In addition, exosome-mediated immune regulation may be associated with adaptive immune responses through toll-like receptors (TLRs) and other receptors. Exosomes mainly induce an innate immune response by identifying specific and conservative endogenous and exogenous molecular patterns of pathogenic microorganisms, causing the release of inflammatory mediators and cytokines, and regulating adaptive immune responses (Fig. 2) [65–67].

Regarding immune-related regulation, exosome-mediated immune regulation may play a role in AP mainly through TLRs. The expression of pro-apoptotic and pro-inflammatory factors were significantly downregulated, whereas the expression of anti-apoptotic proteins and anti-inflammatory factors were upregulated in spinal cord injury models after treatment with BMSC-derived exosomes [68]. Studies regarding PaCa have verified that PaCa-derived exosomes could downregulate TLR4 and downstream cytokines in dendritic cells (DCs) via miR-203 [69]. In addition, TLR-mediated immune regulation can play a role in altering the state of T cells. It has been shown that exosomes derived from adipose tissue-derived mesenchymal stem cells exert ameliorative effects on autoimmune T1DM by increasing the Treg cell population and their products without changing the proliferation index of lymphocytes, which may present a potential approach in T1DM treatment [70].

Exosomes exert strong immunoregulatory effects by regulating the state of immune cells through their immune-active molecules. Among them, TLR4, MyD88, and lysosome-associated membrane proteins have received attention, and their regulatory roles in diabetes, PaCa, and other diseases have been confirmed. The function of exosomes in traumatic pancreatitis remains unknown. After pancreatic trauma, NF-κB is activated to produce various cytokines, such as TNF-α, interleukin (IL)-1β, IL-2, IL-6, and IL-18. These cytokines when released contribute to the production of initial signals, which lead to the infiltration of neutrophils and lymphocytes in the pancreas, thereby amplifying the inflammatory response. Exosomes inhibit the TLR4/MyD88/NF-κB signaling pathway to inhibit NF-κB activation, suggesting that exosome treatment may become a new option for organizational regeneration and repair after pancreatic trauma.

### Exosome-based treatment in angiogenesis

Angiogenesis is a complex process, in which pro- and anti-angiogenic factors coordinate and are generally in a stable state. Once this stabilization is disrupted, the vascular system is activated, resulting in excessive angiogenesis or inhibition of the vascular system, leading to vascular degradation [71, 72]. In recent years, the role of exosomes in promoting angiogenesis has been extensively studied in various disease models. Exosomes can transmit biological information to target cells and alter their biological activities by activating various signaling pathways, including the vascular endothelial growth factor (VEGF) signaling pathway, Shh/Gli signal activation, and release of nitric oxide (NO), leading to vascular protection (Fig. 2) [73–77].

Research regarding the effect of human placental mesenchymal stem cell-derived exosomes on angiogenesis in vitro and in vivo has shown that exosomes promote the formation and migration of human umbilical vein endothelial cells (HUVECs) [78]. A study investigated the specific mechanism by which exosomes enhance the proliferation, migration, and tubular formation of vascular endothelial cells in vitro. These effects may be due to the increased expression of angiogenesis-related molecules FGF-1, VEGFA, VEGFR-2, and ANG-1 [79]. Exosomes can promote angiogenesis by increasing the expression of angiogenesis-related molecules and can regulate angiogenesis by combining with various miRNAs. Another study isolated exosomes from PANC-1 cells and co-cultured them with HUVECs. The results showed that exosomes derived from PANC-1 cells influence angiogenesis in HUVECs in vitro and in vivo by either downregulating or upregulating CCAT1 expression. CCAT1 mediates HMGA1 through competitive binding to miR-138-5p. Overexpression of miR-138-5p antagonized the effect of CCAT1 upregulation on HUVEC angiogenesis in vitro [80].

The effect of exosomes on angiogenesis may have an excellent application in some pancreatic diseases. Traumatic pancreatitis is associated with a relatively high mortality rate, although its incidence is not high in pancreatic diseases. A large amount of pancreatic juice will overflow if there is a severe rupture in the pancreatic duct. Digestive enzymes in the pancreatic juice can also digest pancreatic cells, causing further necrosis of the pancreas and surrounding tissues. Therefore, strengthening blood vessel formation and improving pancreatic microcirculation help delay post-traumatic necrosis and promote the regeneration and repair of pancreatic tissue. Taken together, we speculate that exosomes may promote angiogenesis and provide an alternative direction for research into pancreatic injury tissue regeneration and repair.

### Exosome-based treatment by improving cell migration and proliferation

Under certain pathological conditions, injured and necrotic tissues can form scar tissues through the proliferation and migration of neighboring healthy cells [81]. It is beneficial to stabilize the microenvironment of necrotic tissue, reduce post-traumatic stress, and promote post-traumatic healing. Exosomes are rich in proteins, RNAs, and DNA. These active components can be stably transported between cells, participate in information exchange between cells, and activate the migration and proliferation of various cell types (Fig. 1) [14, 82–86].

In a previous study, human pancreatic cancer Panc-1 cells were subcutaneously inoculated on both sides of the nude mice (1 × 10^6^), and Human umbilical cord mesenchymal stem cells (hUCMSC) exosomes were injected directly into the tumor. It was observed that hUCMSC exosomes promoted the growth of PANC-1 and bxpc3 cells by increasing the proliferation and migration in vitro. In addition, in the xenograft tumor model, hUCMSC exosomes increased the growth of PANC-1 cells [87]. Another study isolated adipose-derived stem cell (ADSCs) from adipose tissue donated by patients and used the supernatant of third-generation ADSCs to extract exosomes. Subsequently, 8 week-old-male Balb/c mice were selected to prepare diabetes models. A full-thickness skin defect of diameter 8 mm was created on the back. Thereafter, 0.2 mL of ADSC exosomes and PBS were injected into the dermis of the experimental group to draw a conclusion. The results showed that exosomes derived from adipose-derived mesenchymal stem cells could enter fibroblasts, promote the proliferation and migration of fibroblasts, and promote wound healing in diabetic mice [88].

When pancreatic inflammatory diseases occur, segmental or diffuse inflammation of the pancreatic parenchyma can lead to pancreatic necrosis, fibrosis, atrophy, and disappearance of acinar and islet cells, resulting in pancreatic structural damage and pancreatic endocrine and exocrine dysfunction. Exosomes stimulate cell proliferation and induce cell activation. Therefore, we speculate that exosomes may present a theoretical basis for the treatment of pancreatic diseases.

## Summary and prospects

Pancreatic disease initiation is a delicate and complex process, and the current management of pancreatic disease focuses on symptom relief and supportive treatment. In recent years, with the development of regenerative medicine, the application of exosomes in the treatment and diagnosis of pancreatic diseases has become an attractive research topic. Exosomes can be obtained from various bodily fluids, and they carry miRNAs that are more sensitive than traditional diagnostic methods, assisting in the early screening of diseases for early treatment and differential diagnosis (Table 1). However, some issues still needed to be addressed before exosomes can be widely used as biomarkers in clinical diagnosis. Firstly, there are large differences in the expression profiles of exosomes in different periods during the dynamic development of pancreatic disease. For example, miR-221 and mir-130a, which are highly expressed in early-stage CP, may serve as diagnostic biomarkers, but in late stage CP, serum-derived exosome expression profiles change considerably [89]. The construction of expression profile for exosomal contents in human pancreatic disease based on dynamic changes will contribute to improved diagnostic accuracy.

**Table 1:** Application of exosomes in the diagnosis of pancreatic diseases

Diseases	Source	Molecules	Mechanism	Years	Ref
AP	Plasma	S100A8 S100A9	Triggered NFκB activation, TNFα	2022	[33]
	PAAF Plasma	miR	High pro-inflammatory activity on macrophages	2019	[34]
CP	Plasma	miR	High exosomal levels of miRNA-10b, miR-21, miR-30c, and miR-181a, and low levels of miR-let7a	2017	[22]
PaCa	Serum	Protein miR	Excellent sensitivity for PaCa versus CP	2015	[39]
	PSC	miR	Stimulated the proliferation, migration, and expression of miRNAs for chemokine ligands	2017	[41]
Diabetes	Urine	Protein	AQP5 and AQP2 were used to classify the clinical stages of diabetic nephropathy	2017	[51]
	Urine	miR	miR-145 may represent a novel biomarker for diabetic complications	2013	[52]

*AP* acute pancreatitis, *CP* chronic pancreatitis, *PaCa* pancreatic cancer, *PAAF* pancreatitis-associated ascitic fluid, *PSC* pancreatic stellate cell

In addition to the expression profile of exosomes, as exosomes are usually isolated from biofluids, determining the source organ of exosomes remains a challenge. Studies have pointed out that lncRNAs/CircRNAs of exosomes exhibit organ/tissue specificity, such as circ-kldhc10 with specificity in exosomes of gastrointestinal origin, and the exploration of specific RNAs carried by exosomes of pancreatic origin will help identify the target organs of serum exosomes [90, 91]. Furthermore, exosomes are usually isolated by applying differential ultracentrifugation under laboratory conditions, and a simple isolation method for exosomes is lacking in hospitals with poor conditions and without laboratories [30]. A study on the validation of exosomes for clinical applications demonstrated a novel and simple method to capture exosomes from blood, which focused on the isolation and identification of exosomes as a simple and highly efficient process via an alternating current electrokinetic chip [92]. The whole blood required for the identification and isolation was only 25 μL, and the process required no longer than 2 h.

Exosomes and their bioactive substances influence cell apoptosis during the development and progression of pancreatic diseases by regulating different signaling pathways, regulating the status of immune cells, and upregulating or inhibiting the expression of related molecules through four main mechanisms (Table 2). Exosomes are smaller, less complex, easier to produce and store than their parent cells, and have no risk of tumor formation. However, exosomes play various roles during different stages of pancreatic diseases, and current research has mainly focused on the complex signals involved in exosome-mediated functional repair and tissue remodeling. We believe that research on exosomes should be longitudinal, multidimensional, and more extensive. Further investigation is required to determine the potential pitfalls associated with the application of exosomes, especially safety, dose–response, and adverse effects. Therefore, research on exosomes is still in the initial stage, and further research is required before their future clinical applications.

**Table 2:** Application of exosomes in the diagnosis of pancreatic diseases

Mechanisms	Diseases	Main research contents	Model	Method of administration	Years	Ref
Apoptosis	AP	Regulate inflammatory cytokines; reduce the infiltration of inflammatory cells; reduce the necrosis of acinar cells; the *NF-κB* signaling pathway	The activation of AR42J pancreatic acinar cells was induced by taurolithocholate treatment	Co-culture	2016	[56]
	Diabetes	Inhibit the apoptosis of rats β cells; alleviate the condition of T2DM	The rat model of T2DM using a high-fat diet and streptozotocin (STZ)	Intravenous injection	2018	[57]
	PaCa	As *miRNA loaders*; Inhibit the proliferation or invasion of PDAC; increase apoptosis and cell cycle arrest	PDAC cells	Co-culture	2019	[58, 59]
			Panc28 cancer cells and the xenograft nude mice model	Co-culture and intravenous injection	2020	
Immune regulation	Diabetes	Increase the number of the *regulatory T cells*; improve autoimmune T1DM; No effect on lymphocyte proliferation index	Streptozotocin-induced of the type-1 diabetes mellitus	Intraperitoneal injections	2018	[70]
Angiogenesis	PaCa	Downregulation/upregulation of CCAT1 from exosomes derived from PANC-1 cells; reduce/enhance the *angiogenesis ability* of HUVECs in vivo and in vitro	PC cells (PANC-1)	Co-culture	2021	[80]
		Overexpression of miR-138-5p; upregulated CCAT1 expression; regulation of *angiogenesis*			2021	[80]
Cell migration and proliferation	PaCa	Promote the growth of Panc-1 and BxPC3 cells; increase the growth of Panc-1 cells in xenograft tumor models	Nude mice were inoculated subcutaneously on both flanks with human pancreatic cancer Panc-1 cells	Administered via intratumoral injection	2021	[87]
	Diabetes	Enter fibroblasts, promote the proliferation and migration of fibroblasts	8-week-old Balb/c male mice were used to prepare a diabetic model	Injected into the dermis	2020	[88]

*AP* acute pancreatitis, *CP* chronic pancreatitis, *PaCa* pancreatic cancer

## Conclusions

Pancreatic diseases are a serious threat to human health and have attracted research attention owing to their uncertain etiology and complex pathophysiology. Exosomes are produced by endocytosis and can be secreted into extracellular vesicles, which participate in cell communication. Exosomes play a corresponding role in the occurrence and development of diseases by regulating cell apoptosis, participating in immune regulation, and promoting angiogenesis, cell proliferation, and cell migration. In addition, the application of exosomes in the diagnosis of pancreatic diseases has become a focus of research. Herein, we summarized the research progress and future challenges associated with exosomes in the treatment and diagnosis of pancreatic diseases. Furthermore, the application of exosomes in pancreatic diseases may provide a sociological benefit in the future.

## Data Availability

Data and materials were available in the manuscript.

## Abbreviations

AP: Acute pancreatitis
BMSC: Bone marrow mesenchymal stem cell
CP: Chronic pancreatitis
DC: Dendritic cell
DN: Diabetic nephropathy
HUVEC: Human umbilical vein endothelial cell
IL: Interleukin
miRNA: MicroRNA
NF-κB: Nuclear transcription factor-κB
NO: Nitric oxide
PAAF: Pancreatitis-associated ascitic fluid
PaCa: Pancreatic cancer
PCS: Pancreatic stellate cell
PDAC: Pancreatic ductal adenocarcinoma
T1DM: Type-1 diabetes mellitus
TLR: Toll-like receptor
TNF: Tumor necrosis factor
VEGF: Vascular endothelial growth factor
